# G-Quadruplex-Based Drug Delivery Systems for Cancer Therapy

**DOI:** 10.3390/ph14070671

**Published:** 2021-07-13

**Authors:** Jéssica Lopes-Nunes, Paula A. Oliveira, Carla Cruz

**Affiliations:** 1 CICS-UBI-Centro de Investigação em Ciências da Saúde, Universidade da Beira Interior, Av. Infante D. Henrique, 6200-506 Covilhã, Portugal; jessicalonu@hotmail.com; 2 Centre for Research and Technology of Agro-Environmental and Biological Sciences (CITAB), Inov4Agro, University of Trás-os-Montes and Alto Douro (UTAD), Quinta de Prados, 5000-801 Vila Real, Portugal; pamo@utad.pt

**Keywords:** G-quadruplex, drug delivery systems, anticancer drugs, drug targeting, nano-particles

## Abstract

G-quadruplexes (G4s) are a class of nucleic acids (DNA and RNA) with single-stranded G-rich sequences. Owing to the selectivity of some G4s, they are emerging as targeting agents to overtake side effects of several potential anticancer drugs, and delivery systems of small molecules to malignant cells, through their high affinity or complementarity to specific targets. Moreover, different systems are being used to improve their potential, such as gold nano-particles or liposomes. Thus, the present review provides relevant data about the different studies with G4s as drug delivery systems and the challenges that must be overcome in the future research.

## 1. Introduction

**Figure 1:**
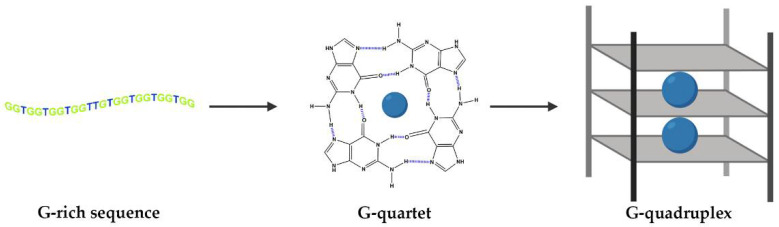
G-tetrads are formed by four guanines bonded via Hoogsteen base pairing. G4s comprise three stacked G-tetrads connected by loops. They are stabilized by monovalent cations coordinated in the central cavity (represented by blue spheres).

DNA and RNA sequences can adopt different conformations, such as duplexes, hairpins and, when they are rich in the guanine (G) nucleobase, they can also fold into G-quadruplex (G4) structures [1]. The building blocks of G4s are G-quartets that are formed through a cyclic Hoogsten hydrogen-bonding arrangement of four guanines with each other. The planar G-quartets stack on top of one another forming four-stranded helical structures. G4 formation is driven by monovalent cations such as Na^+^ and K^+^, and hence physiological buffer conditions favor their formation (Figure 1) [1]. G4 structures present several topologies depending on the cation, strand stoichiometry (intramolecular or intermolecular), polarity, orientation (parallel, antiparallel or hybrid) and loop size (edge-wise, diagonal, double chain-reversal or propeller and V-shaped) [2,3,4].

The G4s oligonucleotides present unique biological features [5]. The majority of the unmodified oligonucleotides present short half-lives in the minute order because they are digested by serum exonucleases and to overcome this issue nuclease-resistant backbones, such as phosphorothioates or 2′- modified analogs, are used [5]. Although, oligonucleotides with the propensity to form G4 structures have increased nuclease resistance, remaining stable for several hours [6,7,8]. Moreover, this tertiary structure demonstrated to be crucial for cellular uptake kinetics. Bishop et al. demonstrated that G4 oligonucleotides were efficiently internalized by HeLa cancer cells, with intracellular levels reaching 5–10-fold above extracellular concentrations [9]. Although, mutations that disrupt this structure led to significantly lower levels of oligonucleotides intracellularly, which was also related to their susceptibility to nucleases [9].

**Figure 2:**
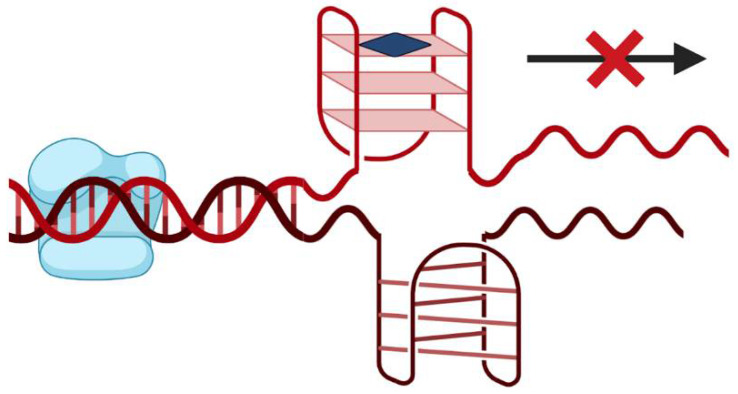
Small molecule (represented by the blue square) driven stabilization of promoter G4s and transcriptional regulation.

G-rich sequences are widely prevalent in human genomes and are present in tandem repeat sequences of telomeric DNA, promoter regions of some oncogenes and within RNA 5′-untranslated regions in close proximity to translation start sites [2,4]. The formation of G4 structures can cause the DNA polymerase arrest [4]. Additionally, G4s located at promoter regions can interfere with transcription factor binding and RNA polymerase activity, thus altering gene expression at transcriptional level [4]. This feature gain interest since it can be used as a potential anticancer therapy [10]. In this sense, several small molecules able to target and stabilize promoter G4 were designed with the purpose to inhibit the transcriptional machinery and down-regulate the expression of target genes (Figure 2). Some of them with G-rich promoter regions and with the ability to fold in G4 structures are c-KIT [11], c-MYC [12], KRAS [13] and BCL-2 [14], which are present in several types of cancers (e.g., pancreatic, melanoma, and other tumors).

More recently, G4s have also been proposed as anticancer drugs, namely by using G4 aptamers. Aptamers are single stranded oligonucleotides that present high affinity to a certain target, which includes proteins, small molecules, ions or even whole cells [4,15]. These nucleic acids can recognize functional domains of proteins, such as catalytic or allosteric sites, thus modulating the biological functions of their targets [15]. They can be immobilized, delivered to a living system or labelled with a functional group without losing their target recognition abilities. Moreover, these molecules were demonstrated to be a good alternative to antibodies, since they present several advantages, such as absence of immunogenicity, higher stability, can be chemically produced with high reproducibility and with less costs and they are also not irreversibly denatured by a variety of solvent and temperature conditions [15,16]. Additionally, G4 structures have a higher negative charge density than duplex DNA, which favors their interaction with cationic proteins or small molecules [4]. These molecules proved that they can be promising anticancer drugs since they have the ability to discriminate between oncogenic and non-oncogenic forms of proteins related with signaling pathways and can be used in in vitro and in vivo assays acting as inhibitors of some oncogenes by blocking their activities [17].

Hitherto there are some aptamers that entered in clinical trials [17]. Some of them present G4 structure and a notable example with several published articles is the AS1411, also known as AGRO100, a DNA aptamer capable to recognize nucleolin (NCL), a protein overexpressed in the surface of several cancer cell types (e.g., gastric cancer, B-cell chronic lymphocytic leukemia, cervical cancer, breast cancer, among others) [6,18,19,20,21]. NCL is over-expressed in malignant tumors and is used as a marker for cell proliferation [15]. AS1411 binds cell surface NCL and inhibits proliferation [5,15]. Nevertheless, a clear molecular mechanism for the AS1411 antiproliferative activity have not yet been fully elucidated. However, potential mechanisms of action for its biological effects in cancer cells are being proposed [5]. The AS1411 aptamer is able to cause biological effects through cell cycle arrest or severely hindered in S phase [22], inhibiting inhibitor of κB (IκB) kinase (IKK) activity, reducing the phosphorylation in response to tumor necrosis factor (TNF)-stimulation [23] and NCL- associated protein arginine methyltransferase 5 (PRMT5) in nucleus, but increases it in cytoplasm, in a dose- and time-dependent manner, playing some role in tumorigenesis [24]. Moreover, AS1411 is able to reduce the binding of cytoplasmic NCL (that plays an important role in regulate the mRNA stability in cancer cells) to blc-2 mRNA in breast cancer cell lines [19]. Additionally, some authors defend that AS1411 is degraded in the presence of serum to products containing guanines, which are toxic to cells (demonstrated in a human acute T cell leukemia cell line, Jurkat E6-1) [25].

AS1411 aptamer was administrated to patients with advanced solid tumors with progressive metastatic in doses up to 40 mg/kg/day (using a four or seven continuous infusion regime) (trial ID: NCT00881244) [5]. Later the clinical trial was restricted to patients with advanced renal cell carcinoma and non-small cell lung cancer [5]. At the end of phase I, promising signs of clinical activity have been reported (multiple cases of stable disease especially for renal cell carcinoma, with all the patients achieving clinical benefits) and absence of severe side effects [5]. Additionally, it was possible to determine maximum tolerated dose or to reach a pre-determined limit for dose escalation, shown that apparently it does not present serious toxicity [5]. Later, in phase II, it was observed that AS1411 has low response rates in unselected patients with metastatic renal cell carcinoma, with dramatic and durable responses and low toxicity (trial ID: NCT00740441) [26,27]. In this clinical trial, one of the patients enrolled presented a dramatic reduction in tumor burden and remained free of progression at least 2 years after completing the therapy [26]. By examining the whole exome sequencing of this patient, it was observed that the tumor presented missense mutations in the fibroblast growth factor receptor 2 (FGFR2) and mammalian target of rapamycin (mTOR) genes [26]. Nevertheless, knowledge along with the other genetic data and nucleolin expression in all patients must be gathered to improve future research in this area. In this sense, AS1411 demonstrated low anti-tumoral efficacy (reaching a maximum plasma concentration of 25.4 µg/mL and concentration causing 50% cell growth inhibition (GI_50_) of about 1–10 µM for most cancer cell lines) and suboptimal pharmacokinetics, being rapidly cleared out from the blood (half-life (t_1/2_)= 1.71 h) [18,26]. Nevertheless, AS1411 has a safe pharmacological profile and specific cancer accumulation, sustaining its study as a potential targeting agent. This aptamer is resistant to 10% serum-containing medium after 3 days [28], although other authors report that the t_1/2_ is about 2 h [25]. 

AS1411 and other G4 aptamers/sequences have been used as a targeting agents/carriers of potential anticancer drugs and functionalized with nano-particles. Thus, in the current review we will summarize several G4-based drug delivery systems that are reported in the literature.

## 2. G-Quadruplex Structures as Drug Delivery Systems

### 2.1. AS1411 as Drug Delivery System

Based on the original promising preclinical properties showed for AS1411, and a good selector for the higher NCL-expressing cancer cells due to its stable G4 structure formed, it could be a good candidate for carrying potential anticancer drugs. In this sense, Shieh et al., used a porphyrin derivative, the 5,10,15,20-tetrakis(1-methylpyridinium-4-yl)porphyrin (TMPyP4), to be delivered by AS1411 [29]. TMPyP4 is a compound commonly used in photo-dynamic therapy (PDT), to treat malignant or premalignant tissues. When excited by optima light, TMPyP4 can generate singlet oxygen in cancer to react with any nearby biomolecules and, thus, promoting cell death [30]. Moreover, TMPyP4 is a G4 ligand, with aromatic and cationic properties, binds G-rich human telomere and inhibits telomerase activity, leading to cellular senescence [31,32]. However, similar to other potential anticancer drugs, TMPyP4 suffers from lack of cancer selectivity and can also damage normal cells [33]. In this sense, AS1411 was used to improve the accumulation of TMPyP4 in breast cancer cells [29]. The authors were based on a previous report in which TMPyP4 was conjugated with the aptamer of prostate-specific membrane antigen (the A10 RNA) [34]. For this purpose, TMPyP4 was deliver through intercalation in the aptamer (without any modification of the molecules) [34]. By using this methodology, it was possible to maintain the efficacy of TMPyP4 and the binding ability of the aptamer [34]. Shieh et al., verified that the complex AS1411/TMPyP4 presented small size, high stability against DNase I digestion, the G4 ligand bind tightly to the aptamer by intercalation and outside binding (binding number of TMPyP4 per AS1411 aptamer was in the range of 5 to 7) and did not disrupt the G4 structure [29]. Moreover, by flow cytometry they demonstrated that free TMPyP4 was more internalized in normal fibroblast and epithelium cells, while the complex was 3.8 times more internalized by cancer cells and the uptake was mediated by NCL [29]. After light irradiation, the complex presented higher photo-damage in a breast cancer cell line than TMPyP4 itself, contrarily to observed in normal epithelium cells [29]. Other studies, also using AS1411 as a noncovalent targeting agent for other anticancer drugs, were performed [35,36]. AS1411 was used to deliver the acridine orange derivative (10-(8-(4-iodobenzamide)octyl)-3,6-bis(dimethylamine) acridinium iodide [C_8_]), which did not negatively affected the recognition of NCL by the aptamer in HeLa cervical cells [35]. The AS1411-C_8_ complex was efficiently internalized by the cells and able to attenuate the toxicity of C_8_ in non-malignant ones [35]. Additionally, the interaction with Zn(II) phthalocyanine (ZnPc) derivatives (ZnPc1-4) [36] was analyzed and it was verified that the compounds presented high affinity to this aptamer (dissociation constant [K_D_] values of 0.09–1.44 µM) and induced a thermal stabilization of 4.3–10.9 °C [36].

AS1411 demonstrated to be applied not only as a drug but also as a transporter of different compounds like acridine [35], phthalocyanines [36] and porphyrin derivatives [29]. Additionally, chemical modifications, such as covalent conjugation to indocyanine green were also performed and the ability of AS1411 to deliver anticancer drugs using a noncovalent approach was retained [37]. 

Additionally, AS1411 modified with FITC (fluorescein isothiocyanate) on its 3′ end, an amine group on its 5′ and six extra T bases to prevent fluorescent (5′-TTT TTT GGT GGT GGT GGT TGT GGT GGT GGT GG TTT TTT-FITC-3′) was used to react with N-heterocyclic carbene (NHC)-Au(I) [38]. The aptamer-NHC-Au(I) conjugate exhibited higher recognition and binding to the target cancer cells (MDA-MB-231, HeLa and DU145 cell lines) as demonstrated by flow cytometry and confocal microscopy [38]. Moreover, by MTS (3-(4,5-dimethylthiazol-2-yl)-5-(3-carboxymethoxyphenyl)-2-(4-sulfophenyl)-2H-tetrazolium) assay, enhanced cytotoxicity was observed when AS1411- NHC-Au(I) conjugate was applied to cancer cell lines and did not significantly reduce proliferation in two healthy normal cell lines (HEK293 and HU1545v) [38]. The authors suggested that this method can be expanded to other metal–ligand constructs, and particular attention should be given to cationic species [38].

**Figure 3:**
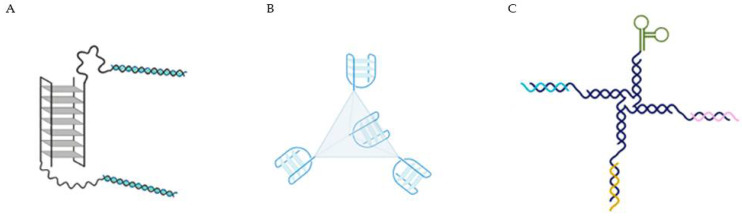
(**A**) AS1411 as a targeting agent in a multifunctional aptamer-based system in which a double stranded (5′-GC-3′ or 3′-GC-5′ base pairs) is used to deliver the doxorubicin [39]. (**B**) DNA tetrahedra structures (DTNs), formed with four molecules of AS1411 bound [40]. (**C**) 4-arm DNA/RNA construct, with one of the arms presenting the AS1411 and the remaining constituted by different siRNA strands (anti-AKT, anti-MDM2 and anti-Survivin) [41].

Other strategies include more complex systems, taking advantage of the targeting properties of AS1411, and including other sequences to act as a drug loading component were done. For example, functional DNA nano-structures were formed by AS1411 as a targeting agent and a double-stranded DNA which is rich in -GC- base pairs to deliver doxorubicin in breast cancer cells (Figure 3A) [39]. The AS1411-dsDNA nano-particle loaded with doxorubicin effectively inhibited the proliferation of resistant breast cancer cells in vitro and in vivo and largely reduced side effect of free drug. These authors concluded that this effect may result from the combination of enhanced permeability and retention (EPR) effects and due to AS1411 aptamer which recognized its target with extraordinary affinity and selectively transported anticancer drug payload into target cancer cells to induce potent cytotoxicity. This methodology was also able to reduce cardiotoxicity frequently related with doxorubicin administration. An alternative strategy relies on the use of DNA tetrahedra structures (DTNs), a 3D molecular cage formed with four DNA chains, that comparably to naked DNA offer greater AS1411 flexibility in design and functionality and probably higher stability in cells (Figure 3B) [40]. In this case, AS1411 was bound to a DTN and administrated in in vitro lung cancer models, where it was observed that DTN itself at different concentrations was not toxic for these cells. Although, when bound to AS1411, it enhanced the cytotoxicity, cellular uptake and induced apoptotic response, intrinsic to this molecule. So, this methodology can be used, taking advantage of the targeting properties of AS1411, to selectively achieve a potential deliver of anticancer drugs. More recently, Tung et al. described a 4-arm DNA/RNA construct, with one of the arms presenting the cancer cell targeting agent AS1411, while the remaining being constituted by different siRNA strands (against protein kinase B (AKT), murine doble minute 2 (MDM2) and Survivin) (Figure 3C) [41]. By using this system, the authors were able to reduce the triple negative breast MDA-MB-231 cell number by about 80% within 24 h with one single administration in the picomoles range, up-regulated the phosphorylation of p53 for more than 8 h, while the three genes of interest were suppressed by nearly half after 4 h of the administration.

### 2.2. Nucleolin Aptamers as Drug Delivery Systems

In order to achieve better structural or antiproliferative results, derivatives of AS1411 are being proposed. AT11, an oligonucleotide derived from AS1411 by an addition of thymine nucleotides to both 5′- and 3′-ends of the DNA sequence and a single G-to-T modification at position 11, resulted in a well-resolved nuclear magnetic resonance (NMR) spectrum consistent with the formation of a single major G4 conformation and in improved anti-proliferative effect [42]. Additionally, other DNA sequences with some AT11 base deletions resulted in an increased thermal stabilization [42]. Since AS1411 can deliver drugs into cancer cells, our group explored the potential of these new sequences as drug targeting agents [6,7,36]. Similarly, AT11, AT11-B0 and AT11-L0 present high affinity to NCL and can selectively deliver potential anticancer drugs, namely acridine and phthalocyanine derivatives to HeLa cancer cells [6,7,36]. Besides the previous described, a new derivative based on the incorporation of a drug in the AS1411 DNA sequence was studied [43]. More specifically, the gemcitabine, a nucleoside deoxycytidine analog and first-line chemotherapy agent for the treatment of pancreatic cancer, was used to substitute the guanine at position 14 and the resulting sequence was designated as APTA-12. This incorporation took place in the loop region of the AS1411 in order to avoid changing of the tertiary structure and protect it from fast degradation by nucleases. Furthermore, this modification allowed us to improve the affinity to NCL, to decrease half-maximal inhibitory concentration and average tumor weight, when compared to AS1411. Later, APTA-12 was conjugated with doxorubicin through a non-covalent strategy [44] and significantly decreased MDA-MB-231 (breast cancer cells) cell viability displaying potential for chemotherapy especially for NCL expressing breast cancer with reduced doxorubicin associated side effects. Another G4 oligonucleotide with affinity to NCL is the RNA sequence found in pre-miR-149, which showed high G4 stability and lower conformational diversity than AS1411 oligonucleotide [45], and it was also able to deliver potential anticancer drugs to prostate cancer cells.

Other AS1411 derivatives, with greater resistance to nucleases can also be used as potential carriers of anticancer drugs. For instance, four new derivatives (AS1411-G, AS1411-GT, AS1411-GT-T8 and AS1411-GT-5′tr) were proposed in order to optimize AS1411 structural features and to find G-rich sequences to improve anticancer properties [46]. Three of them (AS1411-G, AS1411-GT and AS1411-GT-T8) have eight G-tracts, which in principle may allow the formation of two tandem G4s connected by a linker [46]. The four derivatives showed higher thermal stability and inhibitory effect against topoisomerase I than AS1411 [46]. Moreover, most of them (except AS1411-GT-T8) showed an improved anti-proliferative effect on MCF-7 cells compared to AS1411 [46]. Despite a weaker binding to NCL (except for AS1411-GT-T8) [46], AS1411 derivatives could be also an interesting approach to be tested as delivery system for anticancer drugs.

### 2.3. Others Aptamers as Drug Delivery Systems

Apart from NCL, there are other targets that can be used for selective cancer drug delivery, ranging from other surface proteins to whole cells [4,15]. Furthermore, besides the supramolecular strategy, drugs were delivered by other methods. An example is the strategy reported by Kruspe and colleagues [47], in which they used a RNA aptamer, the AIR-3, that specifically binds to the human interleukin-6 receptor in order to deliver a nucleoside analogue, the 5-fluoro-2′-deoxyuridine (5-FUdR). The rationale beyond this system passes through the controlled released of active drugs inside the target cells after the intracellular nucleolytic hydrolysis of the aptamer. For that, 5-FUdR becomes part of aptamer molecule itself, replacing all uridines in the original aptamer. These modifications slightly decrease affinity, although the modified aptamer was still able to bound effectively and specifically to the target receptor. The internalized aptamer is transported to lysosomes, and possible degraded, thus releasing the 5-FUdR, which can be phosphorylated by thymidine kinase within the cell. The resulting 5-fluorodeoxyuridine monophosphate (5-FdUMP) can consequently decline cell proliferation and thus leading to cell death [47]. 

Other G4 aptamers have been developed and besides their anticancer drug delivery properties were not investigated, they can be a potential approach to be used for that purpose. For instance, García-Recio et al. reported anticancer G4 aptamers for MAP kinase interacting kinases (MNK), apMNK2F and apMNK3R, which detected MNK1b protein with high binding affinity (*K*_D_ of 1.79 ± 0.48 and 6.36 ± 1.72 µM, respectively), inhibit translation and decrease 3-(4,5-dimethylthiazol-2-yl)-2,5-diphenyl-2H-tetrazolium bromide (MTT) activity, migration, and colony formation in MDA-MB-231 breast cancer cells, suggesting anti-proliferative effect [48]. Another possibility is the thrombin-binding aptamer (TBA) analogue, TBA535. TBA presents both anticoagulant [49] and anti-proliferative properties [50,51,52,53], but through inversion of polarity sites TBA535 was able to fold in a unique G4 structure and endowed with a better anti-proliferative activity in Calu-6 lung cancer cells but no anticoagulant activity [54]. Additionally, through wound healing assay, the authors shown that both TBA and TBA535 exhibit anti-motility properties [54].

### 2.4. Non-Aptamers G4 Structures as Drug Delivery Systems

Moreover, there are also other non-aptamer G4 sequences that can behave as drug carriers. For instance, Wang et al. developed a bifunctional DNA system composed by a G4 sequence able to carrier TMPyP4, and an aptamer for cellular targeting [55]. In this report, the authors explored two different aptamers, the Sgc8 able to bind to CCRF-CEM cells, a human precursor T-cell acute lymphoblastic leukemia cell line, and TD05 that recognizes B-cell Burkitts lymphoma cell line. The proposed approach presented advantages when compared with the report from Shieh et al. [29]. The new approach with a low drug concentration (10 times less), less incubation time (12 times shorter) and low irradiation energy (7.8 times lower irradiation intensity) [55] showed improved cell targeting and toxicity.

**Figure 4:**
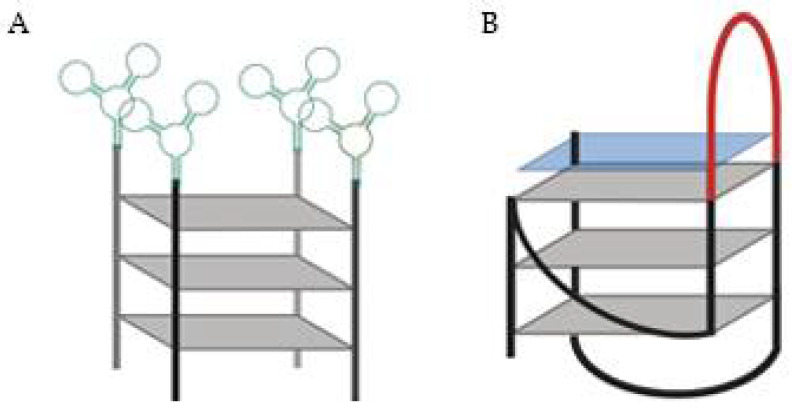
(**A**) DNA aptamer specific for Protein Tyrosine Kinase (PTK)7 (green) linked to 15 consecutive guanines, the AptG15 [56]. (**B**) G4-based drug delivery carriers (GDDC) loaded with an anionic copper(II) phthalocyanine (blue square).The GDDC presents a loop (red), which can hybridize with epidermal growth factor receptor mRNA [57].

Other authors used a DNA aptamer specific for Protein Tyrosine Kinase (PTK)7 linked to 15 consecutive guanines designated by AptG15 (Figure 4A) and it self-assembled into a Y-shaped structure [56]. The four molecules of the aptamer-modified oligoguanine form a G4 structure confirmed by circular dichroism and Raman spectroscopy. The utility of AptG15 as a nano-carrier of therapeutics was tested by loading photo-sensitizer methylene blue to the G4 as a model drug with a loading efficiency of about 84% [56]. The loading ability was due to the G-rich sequences, since when other strategies were used, like the aptamer itself or a C-rich sequence instead, the methylene blue loading efficiency decrease (with loading efficiencies of only 7–6%). The AptG15 showed specific and enhanced uptake to CCRF-CEM cells, which overexpress PTK7 and, with LED irradiation, only the target-expressing cells significantly decreased their cellular viability [56]. 

Another strategy to deliver drugs passes through a G4 sequence being able to recognize and hybridize to a complementary sequence and, consequently releasing a drug that is non-covalently bound to this 3D structure [57]. In this report, G4-based drug delivery carriers (GDDC) were loaded with an anionic copper(II) phthalocyanine, a G4 ligand able to inhibit telomerase activity (Figure 4B) [57]. Moreover, GDDC were designed with a loop, which can hybridize with epidermal growth factor receptor (EGFR) mRNA. Thus, when present in a rich environment in the complementary mRNA, the sequence unfolds and, release the G4 ligand. Additionally, this system can be used as theragnostic strategy, since it can be designed to emit fluorescence when hybridized with the complementary sequence. For that purpose, it is only required that the G4 sequence is labelled in the 3′ and 5′ ends with a fluorescent probe and a quencher. However, the biological potential of this approach remains un-validated since this strategy was only biophysically characterized [57].

## 3. G-Quadruplexes Conjugated with Nano-Particles

One of the biggest roadblocks to develop anticancer drugs is the selective targeting to cancer cells and great part of nano-particles developed take advantage of EPR effect. 

Currently, it is well established that inflammation and hypoxia, which are features of tumoral environments, make vessels more permeable. Consequently, particles ranging from 10 to 500 nm can leave blood flow and accumulate inside interstitial space, by crossing fenestrations in capillaries that can range from 200 to 2000 nm. These particles can be loaded with antineoplastic drugs and consequently be delivered into cancer cells and, ultimately, released from particles. For instance, there is the Food and Drug Administration (FDA)- and European Medicines Agency (EMA)-approved nano-drug Doxil^®^, a liposome loaded with doxorubicin, which is indicated for Acquired Immunodeficiency Syndrome (AIDS)-related Kaposi’s sarcoma, recurrent ovarian cancer, metastatic breast cancer and multiple myeloma [58]. This passive targeting is often used in nano-particles design, since tumors lack from lymphatic drainage [59,60,61]. In normal tissues, the extracellular fluid is constantly drained, allowing for the renewal of interstitial fluid and for sending it back to bloodstream extravasated solutes and colloids. Although, in tumors, the interstitial fluid is poorly uptake, and molecules smaller than 4 nm can return to circulation, while nanoparticles are hindered by their larger size [61].

More recently, it was possible to increase interactions between nano-particles and cells, leading to enhanced internalization of drugs without altering its bio-distribution [61]. To achieve active targeting, specific targets present in cancer cells are being chosen and small molecules, peptides and antibodies, have been widely used to functionalize nano-particles. Nevertheless, when these targeting agents are incorporated in nano-materials, the construct properties are often dominated by the material used than targeting agent, thus being challenging to develop targeted nano-particles with clinical potential. In order to overcome these challenges, aptamers are being proposed and there are some G4 aptamers that were previously referred to that can be interesting agents to be used. Additionally, other G4 sequences can be used as drug loading agents or even help in a controlled release of anticancer drugs [62,63].

### 3.1. Organic Nano-Particles

#### 3.1.1. Liposomes

Liposomes are one of the widest used and successful platforms for drug delivery. They are biocompatible, biodegradable and toxicologically safe. There are some approved therapies based on these nano-particles and clinical trials are also currently in active [64].

Liposomes have been shown to increase the plasma residence time of aptamers. For example, Yi Lu et al. used an aptamer derived from AS1411 (called the NCL-aptamer 5′-GGT GGT GGT GGT TGT GGT GGT GGT GGT TTT TTT TTT TT-cholesterol-3′) and was selected because have high binding affinity to NCL-positive cells. The NCL-aptamer was modified in the 3′-end, with 12 extra T bases to ensure that the binding domain was far away from liposome surface. This modification allows to maintain the binding ability of the aptamer. Additionally, the aptamer was linked to a cholesterol, which was used to immobilize the aptamer on the liposome surface. For that, a solution containing this modified cholesterol was added to dry lipids (HSPC and mPEG2000-DSPE) [65]. It was demonstrated that NCL-aptamer functionalized-liposomes (constituted by HSPC, cholesterol and mPEG2000–DSPE) were capable to deliver cis-diamminedichloroplatinum(II) (cisplatin) a potent chemotherapeutic agent used in a broad range of cancer types that lacks from tumor specificity and presents severe side effects. The NCL-aptamer–liposomes remained predominantly at the cytoplasm of MCF-7 cells, which was consistent with the predicted endocytosis-based internalization mechanism. This endocytosis was NCL-dependent, since NCL-negative cells did not internalize the aptamer-liposomes. By encapsulating cisplatin in aptamer-liposomes, the authors obtained a higher cell damage in NCL-expressing cells, than in negative ones, demonstrating receptor-mediated cell uptake and improved therapeutic efficacy of liposomes that can help to overcome the lack of selectivity of cisplatin. Later, since the in vitro studies demonstrated promising results, the same research group studied the potential of the same NCL-aptamer–liposomes in an in vivo model of breast cancer. For that, they used the aptamer-liposome loaded with doxorubicin, in which they verified that the proposed system was able to penetrate the tumor and decreased its volume/size [66]. Other authors demonstrated a different and efficient strategy to cholesterol tagged NCL aptamer in liposomes composed of DPPC and cholesterol [67]. For that, Nsairat et al. first prepared the liposomes (loaded or not with curcumin) and, then, post-inserted, under stirring, increasing concentrations of cholesterol tagged NCL aptamer [67]. By using this method, the authors were able to avoid that the aptamer ends up in the core of the liposome, maximizing the available space in the core for the loaded drugs [67]. The AS1411 can also be conjugated to liposomes through covalent binding, with an appropriated functional group (NH_2_ or HS) using pegylated-liposomes modified with a functional group (in PEG), such as (COOH or maleimide) [68,69,70,71,72]. This strategy was proposed to target different liposomes formulations (e.g., PEG-PCL, PEG-PLGA, DC-Chol/PEG-DOPE) and it was successfully achieved, allowing to deliver and release the encapsulated drugs. Moreover, for a higher delivery precision, the AS1411-liposomes were conjugated with other targeting molecules such as peptides [68]. For instance, Gao et al., synthesized a dual-functionalized nano-particles with AS1411 and TGN peptide (TGNYKALHPHNG) [68]. With this system, the liposomes first cross the blood brain barrier and, then, deliver selectively the anticancer drug (docetaxel) in brain glioma cells.

Liposomes loaded with anticancer drugs (e.g., doxorubicin) are already available for the treatment of several cancers [58,64]. Although, the release rate is slow (<10% in 24 h). In this sense, triggering mechanisms can be an approach to improve drugs’ release. To overcome this concern, thermoresponsive liposomes were suggested, enabling a local control and rapid trigger drug release [73]. For instance, ammonium bicarbonate (NH₄HCO₃) can be used, which upon mild hyperthermia decomposes rapidly, generating carbon dioxide (CO_2_) bubbles, converting the liposome membranes into permeable defects and yielding a high drug concentration inside the tumor. Hyperthermia can be achieved by ultrasound energy, microwave, radiofrequency, or using magnetic hyperthermia [74]. This treatment is well tolerated by normal tissues, remaining unhurt following treatment for 1 h at temperatures up to 44 °C. Using this rational strategy, Liao et al. produced AS1411-liposomes with doxorubicin and NH₄HCO₃ [73]. In this case, a different strategy to produce AS1411-liposomes was used. Firstly, liposomes composed by DPPC, cholesterol and PEG 2000-DSPE were synthesized and loaded with NH₄HCO₃. Then, AS1411 was conjugated, via formation of a thioether linkage, with PEG 2000-DSPE. The resulting conjugate self-assembled into spherical micelles, which were further incubated with the above-mentioned liposomes (in a 1:5 molar ratio) under sonication, allowing the micelle components to be exchanged into the liposome bilayers [73]. After that, the doxorubicin was loaded in the AS1411-liposomes. As expected, AS1411-liposomes were efficiently internalized by breast cancer cells, contrarily to plain liposomes (without AS1411), after a local hyperthermic stimulus (by using a temperature-controller water mat) [73]. The doxorubicin accumulation in the cell nuclei was markedly increased and the cardiotoxicity associated with the drug was greatly reduced [73].

**Figure 5:**
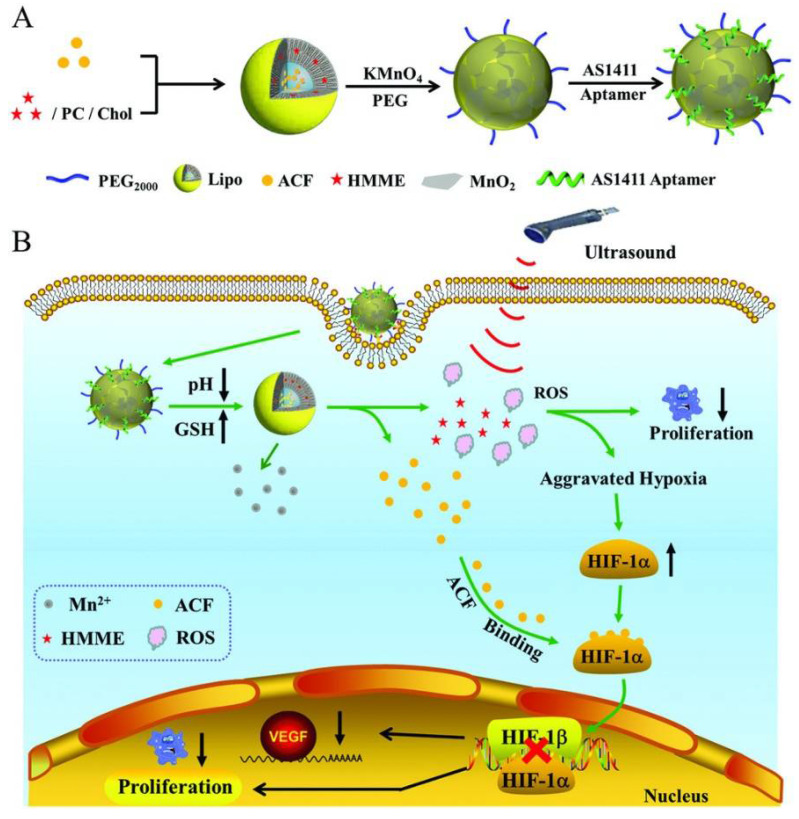
(**A**) Synthesis of Lipo/HMME/ACF@MnO2-AS1411. (**B**) Proposed mechanism of Lipo/HMME/ACF@MnO2-AS1411 for amplification of sono-dynamic therapy. Copyright (2018) Wiley. Used with permission from Wang, L.; Niu, M.; Zheng, C.; Zhao, H.; Niu, X.; Li, L.; Hu, Y.; Zhang, Y.; Shi, J.; Zhang, Z.A. A Core–Shell Nano-platform for Synergistic Enhanced Sono-dynamic Therapy of Hypoxic Tumor via Cascaded Strategy. Advanced Healthcare Materials. John Wiley and Sons, Inc. [75].

Sono-dynamic therapy was proposed to achieve deeper tissue penetration, can cause tumor hypoxia aggravation which can induce malignant cell proliferation and drug resistance. To overcome these disadvantages, Zhang et al. proposed a drug delivery system (Lipo/HMME/ACF@MnO_2_-AS1411) composed by liposomes loaded with acriflavine (ACF) and hematoporphyrin monomethyl ether (HMME) to treat ovarian cancer (Figure 5) [75]. ACF is a specific inhibitor of hypoxia-inducible factor (HIF-1α), which prevents the formation of HIF-1α/HIF-1β dimer, inhibiting the expression of vascular endothelial growth factor and consequently, tumor growth. The HMME can be used a sono-sensitizer and produce reactive oxygen species (ROS) after ultrasound irradiation [75]. The nano-system was also wrapped with manganese dioxide (MnO_2_), which improves mechanical strength of the liposomes to avoid premature drug release before reaching target tissues. Additionally, MnO_2_ can be reduced into Mn^2+^ ions in tumor microenvironment with lower pH value and higher glutathione level, which can be used for drug-controlled release and magnetic resonance imaging. Subsequently, the AS1411 was anchored onto the surface of the MnO_2_ wrapped liposomes for active targeting [75].

The AS1411 targeted liposomes able to trigger an immune response were also developed [76]. In this case, liposomes prepared from cell membranes of red blood cells from rabbit, constituted of cholesterol, phospholipids, and α-Gal glycolipids, were functionalized (through cholesterol modification) with the AS1411 [76] The resulting liposome was able to recognize simultaneously anti-Gal antibodies and NCL overexpressed by tumor cells, followed by activating immune system to attack cancer cells. Thus, resulting in the lysis of cancer cells by antibody dependent cell-mediated cytotoxicity. After tumor environment simulation (in the presence of human blood cells, peripheral blood mononuclear cells, and human IgG), breast cancer cells (MCF-7) treated with the AS1411-liposomes presented a higher lysis rate than cells treated with liposomes without AS1411 [76]. Moreover, in the simulative tumor environment, MCF-7 cells in human blood could be targeted by AS1411-liposomes selectively and the liposomes could recruit the pre-existing anti-Gal antibodies in human blood to the surface of MCF-7 cells to induce the antibody dependent cell-mediated cytotoxicity killing of MCF-7 cells [76].

#### 3.1.2. Micelles

Liposomes are widely used for drug delivery, although there is still some interest in developing micelles, which present a smaller size and can rapidly accumulate in tumor environment [77]. 

Zhang et al. proposed a dual-functional mixed micellar system consisting of an AS1411 decorated with D-α-tocopheryl polyethylene glycol 1000 (TPGS) polymer and pH-sensitive D-α-tocopheryl polyethylene glycol 1000-block-poly-(β-amino ester) (TPGS-b-PBAE, TP) copolymer was established for cancer-targeting delivery and selective cytoplasmic release of paclitaxel in ovarian cancer cells [78]. The enhanced transmembrane ability of this dual-functional mixed micellar system was achieved through AS1411–NCL interaction. The authors observed significantly increased cytotoxicity and G2/M phase arrest against ovarian cancer cells by paclitaxel/dual-functional mixed micelles. Intravenous administration of this system significantly increased tumor accumulation of paclitaxel, inhibited tumor growth, and reduced myelosuppression on tumor-bearing mice compared with free paclitaxel [78]. Moreover, it has been described that the TP copolymer is able to reduce multidrug resistance [79]. Other polymers also demonstrated higher drug release at lower pH environments. For instance, Li et al. used pluronic F127, an amphiphilic polymer, which is associated with poor drug-loading capacity and physical stability, and mixed with beta-cyclodextrin-linked poly(ethylene glycol)-b-polylactide block copolymers (β-CD-PELA)block copolymers, which can self-assemble into micelles with low critical micelle concentration and enhanced drug-loading capacity [80]. By using this hybrid system, they improved micelle stability and drug-loading capacity. These micelles were coated with AS1411 and loaded with doxorubicin, and in acidic pH (pH 5.0) released efficiently this drug (nearly 80%). After intravenous administration, the resulting system displayed long-circulation character in vivo with prolonged circulation time in blood, enhanced accumulation in tumor, improved antitumor activity and decreased cardiotoxicity [80]. 

Furthermore, polymeric micelles with dual pH/redox sensitivity were designed to respond to the acidic tumoral environment and to high concentrations of glutathione (GSH) in intracellular compartments, promoting drug release. Yang et al. developed micelles based on chitosan-ss-polyethylenimine-urocanic acid (CPU) coated with AS1411 [81]. These nano-particles were used to deliver doxorubicin and Toll Like Receptor (TLR)4 siRNA, which affects cancer cells survival, migration and invasion. The administration of AS1411-coated micelles with the two anticancer drugs demonstrated excellent tumor penetration and antitumor efficacy both in lung cancer cell line (A549) spheroids as well as in an in vivo lung cancer model and low toxicity in the systemic circulation.

**Figure 6:**
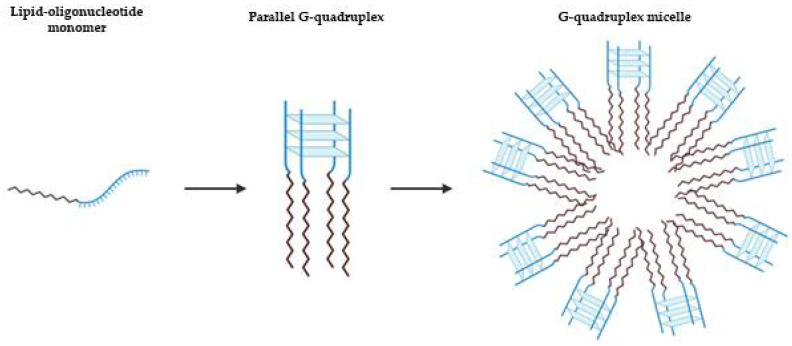
Schematic representation of lipid-driven assembly of tetramolecular parallel G4s. Depending on the nature of the lipid, the sequence of oligonucleotide and of salts present, the tetramolecular parallel G4 can result in micellar assemblies [82].

Other approaches using lipid modification of a G4 prone oligonucleotide sequence were suggested by Vialet et al. to favor the thermodynamics and kinetics of tetramolecular parallel G4 formation over other folds or oligomeric states (Figure 6) [82]. The stability of these micellar assemblies is dependent of the nature of the lipid, the sequence of oligonucleotide and nature of salts present. Similarly, AS1411 with a lipidic tail forms exclusively a parallel G4 structure with a higher stability in a potassium solution than with sodium, and also depending on lipid nature [83]. Based on this rationale, Cozzoli et al. developed a novel and versatile DNA-lipid through amino coupling of 5′-amino-modified DNA strand with lipophilic tails of different length [84]. In the presence of K^+^, the G-rich DNA form a parallel G4, that is essential for the formation and stability of micelles [84]. By hybridization with a complementary oligonucleotide, the micelles were destabilized, resulting in cargo release. In combination with a hairpin DNA aptamer as complementary strand, the release is obtained selectively by the presence of adenosine triphosphate (ATP) [84]. This approach based on G4 micelle disassembly is highly versatile and can be applied to different kind of targets or stimuli.

#### 3.1.3. Other Lipidic Structures

Besides liposomes and micelles, other G4 oligonucleotides-lipidic assemblies were studied.

Prusty et al. developed a version of a hepatocyte growth factor receptor (cMet)-binding aptamer with a lipidic tail and a lipidated GC-rich DNA hairpin loaded with doxorubicin [85]. The DNA modified with lipidic tails self-assemble into a spherical nano-system that recognize cMet [85]. An important outcome of this study was that lipidation of the aptamer motifs leads to a stable nano-construct with high resistance against nucleases, high target-binding affinity, and increased tumor uptake. In addition, the incorporation of 2′,6′-dimethylazobenzene loaded with the doxorubicin enabled the release of anticancer drug by photo-isomerization [85]. These authors suggest that this platform can be used for simultaneous delivery different regulatory molecules, such as siRNAs, microRNAs, anticancer drugs, and other molecules with higher specificity to prevent functions of disease-relevant biomolecules.

**Figure 7:**
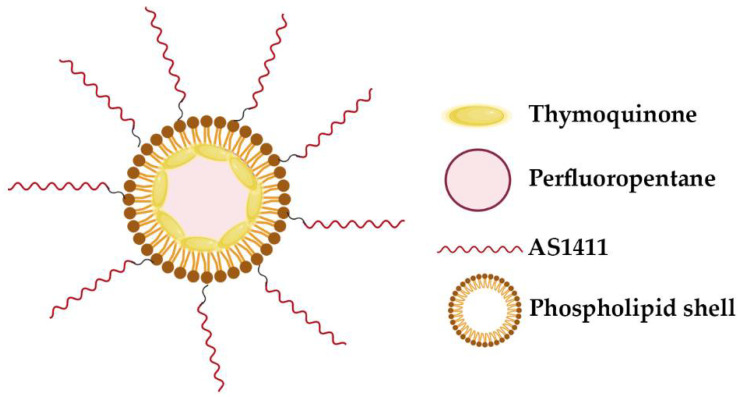
Schematic of AS1411-conjugated nano-droplets loaded with thymoquinone. The perfluorocarbon-based nano-droplet emulsions were coated with a phospholipid shell, formed by DPPC/DSPE-PEG2000 [86].

Other strategy relies on G4-lipidic nano-particles achieved through the use of biocompatible perfluorocarbon-based nano-droplet emulsions encapsulated by a phospholipid shell, formed by DPPC/DSPE-PEG2000 (Figure 7) [86]. When ultrasound is applied, perfluorocarbon undergoes to a phase change and induce transient perforation of membranes in close proximity. This phenomenon is known as sono-poration and allows for the enhanced intracellular delivery of drugs. Murphy et al. evaluated AS1411-conjugated nano-droplet emulsions for targeted delivery of molecular compounds to cancer cells. In this study they loaded thymoquinone, a natural hydrophobic phytochemical compound with bioactivity in cancer cells, in AS1411-conjugated nano-droplets and investigated their effect on human breast cancer cells [86]. By confocal microscopy, it was observed that the AS1411-conjugated nano-droplets were internalized by breast cancer cells and significantly enhance uptake and cytotoxicity in cancer cells compared to free thymoquinone. This formulation can be expanded to other hydrophobic drugs and may offer significant potential for targeted delivery of antineoplastic drugs to tumors for cancer treatment.

Other two systems were proposed by Vandghanooni and colleagues to be used in cisplatin resistant ovarian cancer cells [87,88]. In one of the proposed approaches, AS1411-decorated PEGylated poly(lactic-co-glycolic acid) nano-particles containing cisplatin (Ap–CIS–NPs) or anti-miR-21 (Ap-anti-miR-21-NPs) were prepared [87]. After nano-particles characterization, the biological potential was assessed and, for that, cisplatin resistant A2780 cells (A2780 R) were first treated with Ap-anti-miR-21-NPs to decrease the drug resistance and sensitize the cells to cisplatin [87]. Afterward, miR-21-inhibited cells were exposed to the Ap–CIS–NPs. This strategy resulted in enhanced mortality in the miR-21-inhibited cells after the delivery of cisplatin using Ap–CIS–NPs [87]. Later, Vandghanooni et al. prepared star-shaped glucose-core polycaprolactone-polyethylene glycol (Glu-PCL-PEG) block copolymer containing cisplatin (CIS-PCL NPs) and locked nucleic acid (LNA) anti-miR-214 (LNA-PCL NPs) [88]. The resulting nano-particles were decorated with AS1411 to improve their internalization by ovarian cancer cells [88]. As performed in the previous work, cells were firstly treated with LNA-PCL NPs to inhibit oncomiR-214 and sensitize the cells to cisplatin. Next, the miR-214-inhibited cells were exposed to the Ap-CIS-NPs and caused enhanced apoptosis, which was further confirmed by apoptosis detection and evaluation of downstream genes expression [88].

Niosomes are self-assembled vesicles made up of single chain non-ionic surfactants combined with appropriate amounts of cholesterol or other lipids, exploited as carriers for hydrophilic or lipophilic drugs [89]. The drug is entrapped in the membrane due to the self-assembly of the surfactant and lipid molecules, generally organized in stable bilayers [89]. These systems are typically more stable, less expensive than liposomes. Riccardi et al. developed niosomes loaded with nucleolipidic Ru(III)-complex HoThyRu, an anticancer agent, and decorated with the AS1411 aptamer, allowing selective recognition of cancer cells [89]. The niosomal formulations were prepared from 2,3-bis(tetradecyloxy)propan-1-aminium chloride and the non-ionic surfactant polysorbate 80 [89]. These formulations were tested on both cancer and normal cells and showed promising anti-proliferative activity on HeLa cells [89] AS1411-decorated niosomes as effective nano-carriers for Ru(III)-based drugs in anticancer strategies. Notably, AS1411 proved to markedly enhance the bioactivity of the Ru(III)-containing niosomes, since per se did not cause any cytotoxic effect at all the tested concentrations (1.25, 3.5, 7.5, 25 and 50 µM) after incubation for 72 h [89].

#### 3.1.4. Dendrimers

**Figure 8:**
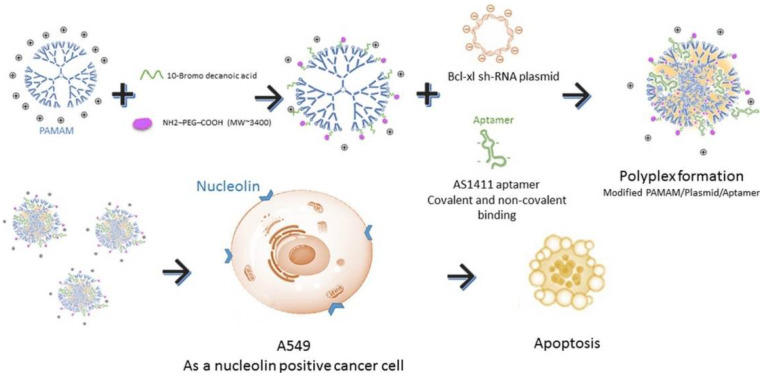
Schematic representation of AS1411-conjugated modified PAMAM dendrimers. These dendrimers efficiently target nucleolin positive cells (A549 cells), knockdown the protein expression of Bcl-xL and induce apoptosis in the target cells. Reprinted from The International Journal of Biochemistry and Cell Biology, 92, Ayatollahi, S.; Salmasi, Z.; Hashemi, M.; Askarian, S.; Oskuee, R.K.; Abnous, K.; Ramezani, M., Aptamer-targeted delivery of Bcl-xL shRNA using alkyl modified PAMAM dendrimers into lung cancer cells, 210–217, Copyright (2017), with permission from Elsevier [91].

More recently, dendrimers are also used as drug delivery systems, which can be covalently bound to G4 aptamers with the aim to improve their cancer targeting [90]. Dendrimers are hyper-branched polymers consisting in series of repetitive units extending outward from the core [90,91,92]. They can have a precise molecular structure, high geometric symmetry, controllable molecular weight which contains an internal cavity for macromolecules encapsulation and also free functional groups on their surface that allow aptamer functionalization for targeted delivery [90,91,92]. This strategy was already used to deliver anticancer drugs (epirubicin [93], camptothecin [90] or doxorubicin [92]) or even plasmid-based short hairpin RNA (shRNA) [91] and was also used as theragnostic nano--drug delivery system [92]. Overall, these systems were able to improve the toxicity of anticancer drugs and enhance the therapeutic efficacy, with an efficient drug loading tumor targeting [90,93] and in some cases also pH-dependent drug release [92,93]. Additionally, aptamer-functionalized dendrimers shown high transfection efficiency, low cytotoxicity and Bcl-xL selective inhibition of cancer cells [91] For instance, AS1411-conjugated modified polyamidoamine (PAMAM) dendrimers efficiently target NCL positive cells (A549 cells), knockdown the protein expression of Bcl-xL and induce apoptosis in cancer cells (Figure 8) [91]. These results indicated that the AS1411 aptamer conjugated vector-shRNA complex could be used in further studies for gene therapy specially for pulmonary systems with great features including high transfection efficiency, low cytotoxicity and selective inhibition of cancer cells.

### 3.2. Inorganic Nano-Particles

#### 3.2.1. Gold Nano-Particles

Gold-nano-particles (AuNPs) are forerunners for the development of a therapeutic delivery vehicle [94]. Reasons for this include their low toxicity, optical properties and ease of functionalization [94,95]. Many studies have shown that AuNPs modified with oligonucleotides exhibit high cellular uptake and internalization without the use of additional transfection reagents through enhanced binding of class A scavenger receptors on the cell surface [94]. 

Furthermore, the dense layer of oligonucleotides on AuNPs allows them to become more resistant to degradation and increases the stability when compared to the oligonucleotide without the nano-particle [95]. However, upon intravenous administration, AuNPs encounter several serum proteins that often form a corona around them [96]. Especially, the binding of opsonin proteins leads to recognition and, subsequently, sequestration by macrophages, decreasing the nano-particles residence time in the bloodstream and decreasing its accumulation in the target tissue. Although, AuNP core spherical nucleic acid with a 3′ thiol-modified guanine-rich sequence that forms G4s alter the chemical composition of the spherical nucleic acid protein corona, increasing the macrophage uptake which cause increased clearance from blood stream, allowing different bio-distribution and pharmacokinetic profiles in vivo [96]. However, in different in vitro experiments, the G-rich DNA-modified AuNPs are highly internalized by cancer cells comparably to A, T, or C-rich DNA modified AuNPs and can efficiently deliver antineoplastic drugs [97]. 

**Figure 9:**
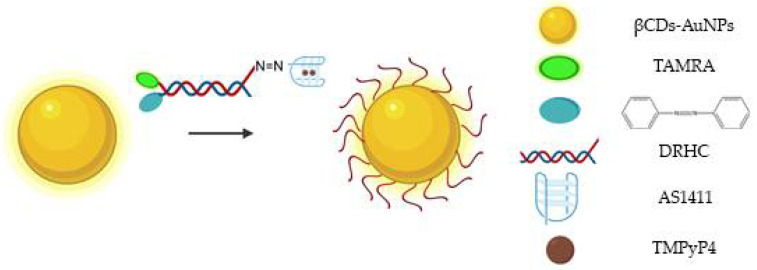
Schematic representation of AS1411-functionalized βCDs-AuNPs, which also contain: hypoxia-induced cleaved azobenzene bridges, DRHC tagged with TAMRA and the photo-sensitizer TMPyP4 [100].

Due to their physical and chemical properties, AuNPs have been functionalized with drugs, namely, aptamer AS1411 to improve cellular uptake and anti-proliferative/cytotoxic effects [98]. Indeed, Malik et al. showed its antitumor efficacy in an animal model of breast cancer when compared to unconjugated AS1411 or AuNPs linked to control oligonucleotides, and the cancer selectivity was maintained [98]. Additionally, it can be administrated for targeted imaging and efficient cancer cells PDT. Some hydrophobic photo-sensitizers have poor or limited solubility in aqueous solutions, reducing the yields of ROS able to destroy cancer cells, and, consequently, leading to nonspecific photo-damage of normal tissue. To overcome this drawback, AuNPs functionalized with AS1411 were synthesized and conjugated with a porphyrin derivative for targeted imaging and efficient PDT [99]. This strategy enables an efficient internalization by NCL-expressing cells and, after light illumination, the photo-sensitizer was able to produce ROS and decreased the cell viability [99]. Combinatory therapies achieved by PDT and gene therapy were also delivered by AS1411-functionalized AuNPs (modified with β-cyclodextrins, βCDs), which also contained: hypoxia-induced cleaved azobenzene bridges, HIF-1α-against antisense oligonucleotide (ASO)/G4-constituted double-stranded DNA/RNA hybridization complex (DRHC) and the photo-sensitizer TMPyP4 (Figure 9) [100]. After light irradiation, the TMPyP4 was activated, consumed oxygen and potentiated hypoxia. After that, the hypoxia-responsive azobenzene bridges were reduced by highly expressed reductases to amines under the oxygen environment low level, which triggered the ASO release and allowing a synergistic therapy with PDT for tumor growth inhibition [100]. The released ASO was able to silence HIF-1α under PDT-induced hypoxia, and thus achieved hypoxia-triggered gene therapy [100].

**Figure 10:**
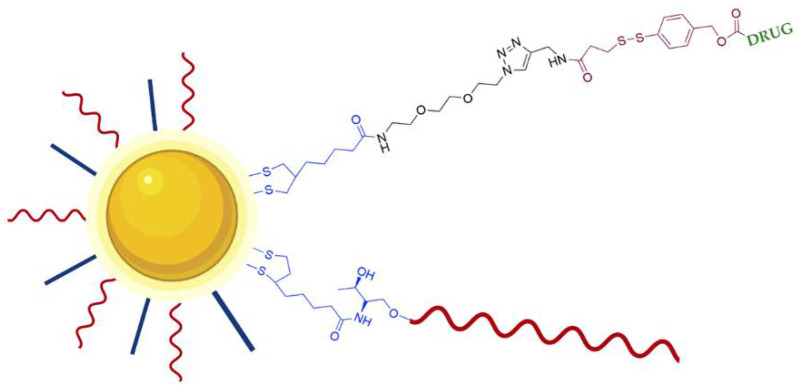
Schematic representation of DNA stabilized GNPs loaded with drug (Doxorubicin or AZD8055). AS1411 (red line) is conjugated through a dithiolane linker (blue). The drug is conjugated through a bifunctional linker composed of an anchoring dithiolane moiety (blue) and self-immolative fragment (brown). The release of the drug is triggered by glutathione breaking the disulfide group present in the self-immolative fragment [102].

In order to improve the efficacy of AS1411-AuNPs, strategies to enhance the loading density of AS1411 were studied, and it was demonstrated that the solution used can influence this process [101]. For instance, low pH solution can increase up to 2.5 times the AS1411 loading efficiency than the conventional salt-aging process, and this can result in an improved uptake as well as a higher in vitro efficacy of the nano-constructs in cancer cells [101]. Although, other modifications can be performed to improve the response rate of AuNPs and reduce side effects relatively to traditional therapeutics. For example, Latorre et al. developed AS1411 modified AuNPs loaded doxorubicin or AZD8055 (small molecule inhibitor of Phosphoinositide 3-kinase [PI3K] and mTOR isoforms). Doxorubicin and AZD8055 were attached to AuNPs using a bifunctional linker containing a dithiolane and a self-immolative disulfide-based structure. Dithiolane allows attachment of drugs onto the gold surface and the self-immolative fragment facilitates the release of drugs upon an intracellular stimulus (Figure 10) [102]. The release of doxorubicin and AZD8055 is triggered by the higher concentration intracellularly of glutathione. The use of dithiolane moiety presented two advantages relatively to other thiol groups that are commonly used for the DNA-gold nano-particles preparation which are the absence of the removal of thiol-protecting groups from the oligonucleotides and the binding is faster and the stability of the resulting nano-particles is increased. The authors showed that these modifications increase targeted delivery of therapeutics with AuNPs, reducing cell viability in breast cancer and uveal melanoma cell lines [102].

#### 3.2.2. Graphene Nano-Particles

Graphene oxide (GO) is a carbon-based nano-material with a greater biocompatibility compared to heavy metal-based inorganic ones [62]. It enables modification with biopolymers on their surface through different functional groups and can be degraded by peroxidase enzymes, suggesting its biodegradability in specific microenvironments [103]. These modifications on a surface of GO are highly desired to achieve degradation after its biological function (i.e., drug delivery), especially at complex in vivo level [62]. However, such modifications limit the degradability of GO due to the steric hindrance and consequently limited activity of enzymes on its surface [62]. 

**Figure 11:**
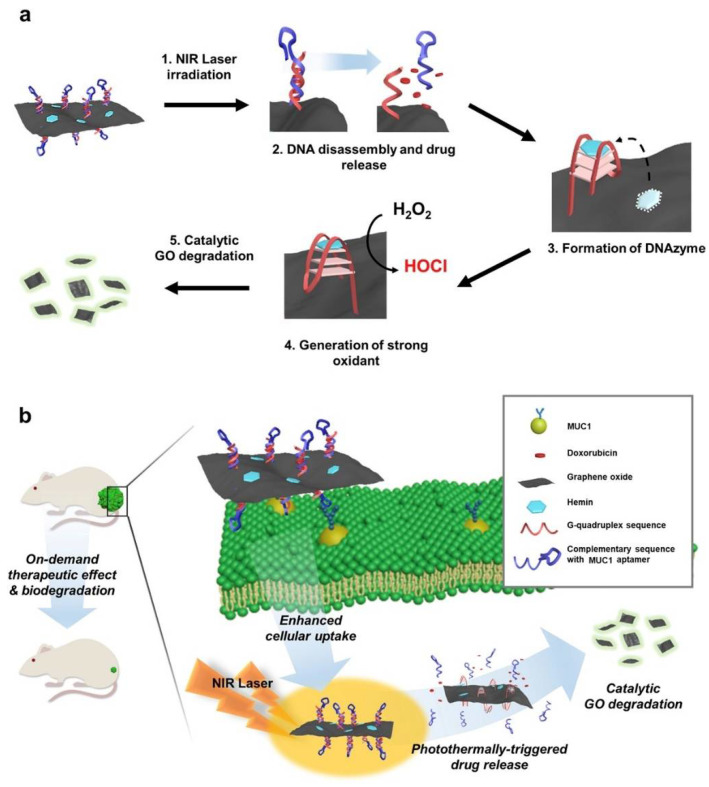
Schematic illustration self-catalytic GO-He-G4-M@DOX nano-system for cancer therapy conducted by a DNAzyme and controlled by photo-switch. (**a**) Design of GO-He-G4-M@DOX operated by photo-switch. (**b**) The systemic application of GO-He-G4-M@DOX can result in the controlled anticancer release and, subsequent, biodegradation into fluorescent small fragments of GO. Reprinted from Biomaterials, 263, Lee, H.; Kim, J.; Lee, J.; Park, H.; Park, Y.; Jung, S.; Lim, J.; Choi, H.C.; Kim, W.J., In vivo self-degradable graphene nano-medicine operated by DNAzyme and photo-switch for controlled anticancer therapy, 120402, Copyright (2020), with permission from Elsevier [62].

Due to the ability of some stimuli such as pH, ions and biomolecules to alter the conformation of some DNA sequences, they are an interesting functional domain. For instance, single stranded G4 sequence on the surface of GO forms a quartet structure and becomes DNAzyme by binding with hemin on the GO surface, exhibiting peroxidase effect [62]. In this sense, Lee et al. took advantage of functional DNA and GO features and developed a self-catalytic GO-He-G4-M@DOX nano-system for cancer therapy conducted by a DNAzyme and controlled by photo-switch (Figure 11) [62]. 

The carboxylic acid of the GO was conjugated with amine-modified DNA, composed by a G4 sequence and its complementary sequence with Mucin1 aptamer for targeted delivery [62]. Additionally, by intercalation, doxorubicin was loaded on the DNA double strand, whereas hemin was loaded on the surface of GO [62]. After internalization of GO nano-composite and turning on the photo-switch, the doxorubicin was released by the transition of DNA double strand into single stranded, which was triggered by photo-thermal effect of GO [62]. After that, the single stranded formed a quartet structure, and hemin bound to it, inducing a catalytic peroxidase effect. Due to the high concentration of oxygen peroxide in cancer cells, the G4-hemin complex generate hypochlorous acid (a strong oxidant) and self-degrade GO into fluorescent small fragments for potential clearance [62].

#### 3.2.3. Silica Nano-Particles

Mesoporous silica nano-particles are interesting biocompatible drug delivery systems able to store and release molecules [104]. They can release drugs through different stimuli-responsive mechanism [63]. Although, some endogenous stimuli can cause an unpredictable activation and limit the drug delivery at the desired regions [63]. Thus, for therapeutic applications, a release mechanism induced by a physical stimulus can be advantageous [63]. For instance, light induced release is an attractive option, but some system can present some disadvantages since they have limited light tissue penetration and can induce high toxicity in non-malignant tissues [63]. In this sense, photo-sensitizer agents can be used in PDT to treat the target tissues after irradiation with light at specific wavelengths that can achieve deeper regions [63].

**Figure 12:**
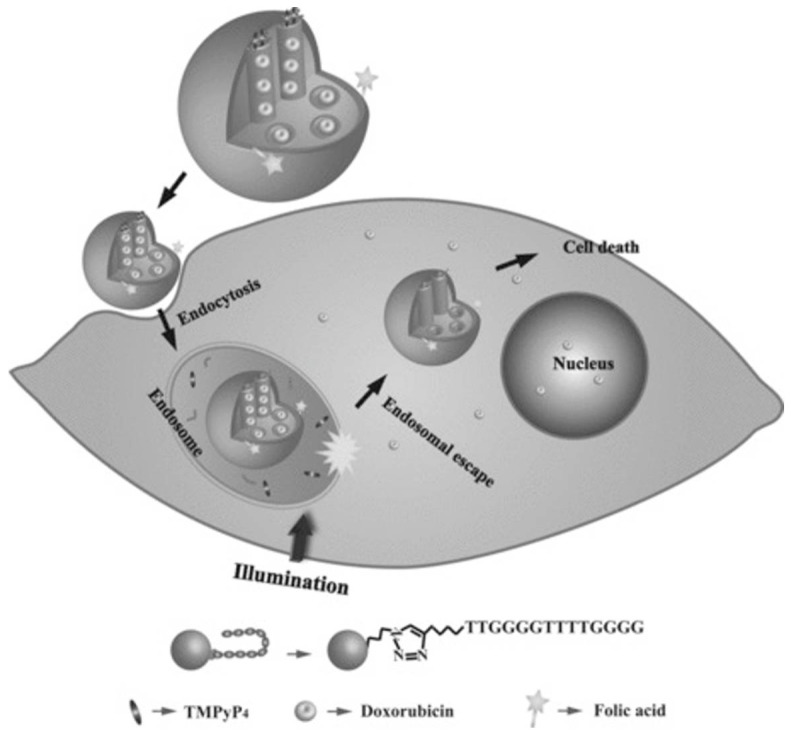
Representation of a novel nano-system for targeted intracellular drug delivery composed by photo-sensitizer-incorporated G4 DNA-capped mesoporous silica nano-particles. This system is composed by a photo-sensitizer (TMPyP4) that upon light irradiation generates ROS, leading to cleavage of G4 DNA ((G_4_T_4_G_4_)_2_) capping agent and allowing doxorubicin release. The nano-system is internalized via folate receptor-mediated endocytosis. Copyright (2013) Wiley. Used with permission from Chen, C.; Zhou, L.; Geng, J.; Ren, J.; Qu, X. Photo-sensitizer-incorporated quadruplex DNA-gated nano-vechicles for light-triggered, targeted dual drug delivery to cancer cells. Small. John Wiley and Sons, Inc. [63].

Taking this into account, Chen et al. created a novel nano-system for targeted intracellular drug delivery composed by photo-sensitizer-incorporated G4 DNA-capped mesoporous silica nano-particles [63]. Since this system is composed by a photo-sensitizer (TMPyP4), upon light irradiation, it generates ROS, leading to cleavage of G4 DNA ((G4T4G4)_2_) capping agent and allowing drug release. Moreover, besides the capping agent function, the G4 DNA also acts as a carrier of TMPyP4 (Figure 12). This nano-system is a synergistic combination of chemotherapy and PDT for cancer treatment with spatial and temporal control [63]. Additionally, through the introduction of folic acid, they obtained tumor targeting efficiency [63], since the folate receptor is upregulated in different cancer cells [105], resulting in a specific internalization of the nano-system by the cancer cell line and localize the endolysosomal compartment [63]. The authors also suggest that the light excitation might induced endosomal membrane damage mediated by ROS produced by the TMPyP4 [63]. Additionally, to test the anticancer drug delivery, this nano-system was used to deliver doxorubicin into cancer cells and it was verified that the complete nano-system (composed by the mesoporous silica nano-particles with the G4 DNA, the TMPyP4 and the folic acid loaded with doxorubicin) exposed to light irradiation presented higher cytotoxicity than all tested control situations (except when compared to free doxorubicin, which has a slightly higher toxicity) [63]. This effect was probably caused by the limited illumination time that could be unable to complete uncap the DNA and, thus, incapable to release the doxorubicin from the mesopoporous silica nano-particles, while the free drug can easily diffuse through the cells. However, the authors claimed that this approach could be more efficient to apply in vivo, due to the targeting properties of the folic acid, avoiding nonspecific effects in nonmalignant regions [63].

#### 3.2.4. Magnetic Nano-Particles

Magnetic nano-particles can be organic or inorganic and present some interesting features since they can be guided through a magnetic field, heated, and thus triggering drug release or to producing hyperthermia/tissue ablation and also superparamagnetic nano-particles can be visualized by magnetic resonance imaging [106]. The most used bulk materials for magnetic applications are iron oxide (Fe_3_O_4_) and manganese ferrite (MnFe_2_O_4_) [107]. Coating materials used for magnetic nano-particles include polyaniline, polyisobutylene, polyethylene-based polymers such as poly(ethylene-graft-tetraethylenepentamine maleimide) and polystyrene-based polymers such as poly(styrene-graft-tetraethylenepentamine) and poly(styrene-block-tetraethylenepentamine) [107]. For example, the core–shell structure of Fe_3_O_4_-polyaniline has an average diameter of 80 nm, with the polyaniline shell to provide conductive feature [108]. These nano-particles can be used as magnetic drug delivery vehicles and for imaging-guided delivery. Indeed, it can be attached with cytotoxic drugs which, after intravenous or intra-arterial injection, can be guided to tumors through a magnetic field [106]. After accumulation of the carrier in the target region, the drug can be released through different stimuli, such as enzymatic activity, pH, osmolality or temperature [106]. In theory, this strategy offers the ability to deliver the drug to a specific region, enhancing the uptake and thus requiring lower doses and reduce potential systemic toxicity. Although, the major drawback is the damage of normal cells caused by the magnetic field [106]. 

In this sense, the surface functionalization with an aptamer able to perform tumor targeting was employed by Sun et al., which developed superparamagnetic iron oxide nano-particles (SPION) functionalized with AS1411, to increase the affinity and specificity to cancer cells [106]. Moreover, the authors take advantage of the carrier ability of AS1411 and loaded it with porphyrin photo-sensitizer TMPyP. Additionally, they decided to conjugate an anticancer drug, the daunomycin, an anthracycline that is widely used in various cancers (namely in Kaposi’s sarcoma), bound to a duplex DNA strand (a GC-rich and hairpin-forming DNA, the S8). The clinical use of this anticancer drug is restricted by dose-dependent toxicity, multidrug resistance and low specificity [109]. So, daunomycin was used in this cancer-targeted nano-carrier to overcome some of the drawback of this drug. After synthesis of the SPION nano-particles functionalized with a DNA hybrid sequence (that include the AS1411 and the S8) for accurate targeting drug delivery and chemo-photo-dynamic therapy, the stability in serum and targeted drug delivery to cancer cells were analyzed [106]. Firstly, it was confirmed that drugs were efficiently loaded in the AS1411-S8 and the nano-system exhibited good stability in serum and nuclease DNase I and can be administrated in the bloodstream. The SPION functionalized with the AS1411-S8 loaded with the drugs were successfully internalized by a lung cancer cell line (A549 cells), efficiently entered into the nucleus of cells, and it was demonstrated that when applied a magnetic field, they migrate to this region and the cells tend to die [106]. Moreover, visible light irradiation could dramatically increase the ROS production of the photo-sensitizer TMPyP [106]. Furthermore, an NCL blocking experiment indicated that AS1411 played an important role in enhancing the targeting accuracy of the drug delivery system [106]. In summary, the proposed system has high anticancer efficiency and exhibited great potential in the era of precision medicine.

### 3.3. Other Types of Nano-Particles

**Figure 13:**
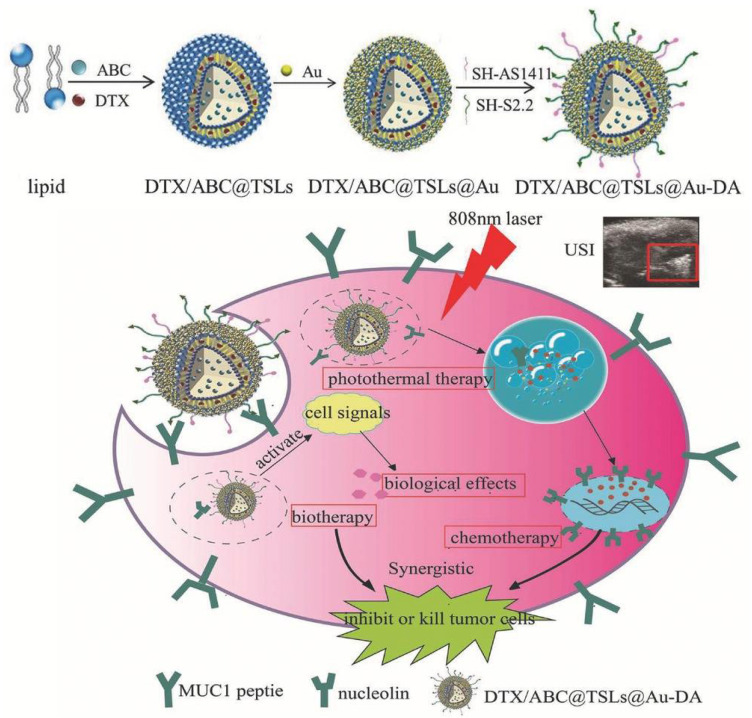
Schematic representation of a near-infrared photo-thermal responsive dual aptamers-targeted docetaxel-containing nano-particles. In this system, docetaxel and NH_4_HCO_3_ were loaded in liposomes. The resulting liposomes were subsequently coated with gold that produce heat under 808 nm laser irradiation to induce nano-particles’ decomposition. through thermal activation, the NH_4_HCO_3_ containing thermosensitive liposomes generate CO_2_ bubbles and the acidic environment at the tumor region that promote bubbles generation. The thermosensitive liposomes were then functionalized with nucleolin and Mucin1 peptide aptamers, the AS1411 G4 and S2.2 sequences, respectively, for a specific internalization by MCF-7 cells. Copyright (2017) Wiley. Used with permission from Zhao, F.; Zhou, J.; Su, X.; Wang, Y.; Yan, X.; Jia, S.; Du, B. A Smart Responsive Dual Aptamers-Targeted Bubble-Generating Nano-system for Cancer Triplex Therapy and Ultrasound Imaging. Small. John Wiley and Sons, Inc. [110].

Besides all the above-mentioned nano-particles, there is still some other strategies that result from a mixture of organic and inorganic components. For example, Zhao et al. designed a near-infrared photo-thermal responsive dual aptamers-targeted docetaxel-containing nano-particles (Figure 13) [110]. In this system, docetaxel and NH_4_HCO_3_ are loaded in liposomes. However, since local heating is often difficult to achieve in clinical applications, liposomes were coated with gold that produce heat under 808 nm laser irradiation to induce nano-particles’ decomposition [110]. Moreover, through thermal activation, the NH_4_HCO_3_ containing thermosensitive liposomes can generate CO_2_ bubbles and the acidic environment at the tumor region could also promote bubbles generation. The CO_2_ bubble production can disrupt cell membranes and enhance drug release [110]. The thermosensitive liposomes were then functionalized with NCL and Mucin1 peptide aptamers, the AS1411 G4 and S2.2 sequences, respectively [110]. Both, NCL and Mucin1 peptide, are overexpressed in cancer cells surface, which leads to a higher cancer targeting efficacy, avoiding an inefficient delivery due to receptor saturation. The resulting nano-system presented good biocompatibility and uniform size (diameter about 200 nm) [110]. Moreover, after 808 nm laser irradiation, the drug loaded was successfully released (84% at 4 h) [110]. Relatively to the biological potential, after in vitro and in vivo experiments, the nano-system demonstrated that it can synergistically inhibit tumor growth by combination of chemotherapy, photo-thermal and biological therapy [110]. The dual ligand functionalization increases cellular uptake on breast cancer cell line cells and achieves ultrasound imaging (USI) at tumor site [110]. Overall, the nano-system was demonstrated to be a promising theragnostic system achieved by a combination of different antineoplastic drugs.

## 4. Conclusions

The delivery of conventional chemotherapies can cause several side effects, including systemic toxicity and reduced immunity and can damage other organs, namely the heart and kidneys. Thus, selective targeting of malignant cells is a critical step that can mitigate some off target effects.

G4 has been studied and increasingly applied in nano-technology because of its distinctive G4 stranded structure which provides the potential for self-assembly, conformational switching, ligand binding and so on. The rational design of ligands to selectively interact, stabilize or cleave G4 structures is a promising strategy to serve as anticancer drugs in the medical field [111]. G4 structures are also able to carry anticancer drugs and its conjugation with certain nano-particles increase the loading ability and, consequently, potentiate biological effects [112]. In addition, as the above examples have shown, some aptamers can intrinsically adopt a G4 structure, which enables extra targeting. AS1411 is the most used G4 sequence as a drug carrier whether complexed or not with nano-particles. Relative to G-rich sequences, it was demonstrated that sequences able to form parallel G4 (e.g., PU22, T40214 and AS1411) were mainly detected in the lysosome and were retained in cancer cells, while the ones that do not form G4 were mainly found in the mitochondria and likely distorted after cellular uptake [113], thus demonstrating that the design and the rationale under a G-rich sequence is an important step for drug delivery systems conceptualization [113].

However, some drug delivery systems, which present very promising results in in vitro experiments, do not necessarily need to be translated in the best candidates for therapeutic studies [114]. Hence, in vivo experiments are fundamental to evaluate clinical relevance and they were performed in several studies to evaluate the anticancer potential of G4-based drug delivery systems (Table 1 and Table 2). For instance, nano-particles with less than 10 mm can be rapidly filtered by the kidneys and with diameter between 10 and 200 nm are mainly retained in the liver and spleen [114]. The charge is also a critical issue since nano-particles positively charged can disrupt the cell membrane and negatively charged can accumulate in mononuclear phagocyte system, are less toxic and are prone to opsonization [114]. Moreover, nano-particles that can present similar uptake and in vitro response in multiple cell lines, can act differently in in vivo tumor models, which can be related with angiogenesis, necrotic regions, and macrophage abundance levels in different models [114].

Over the years G4-based delivery systems have been proposed and tested, demonstrating a promising approach to treat different types of cancer. These systems present high selectivity to malignant cells and are able to decrease the major chemotherapies’ side effects. Although, in some cases biological characterization failed since the in vitro response was very different from the in vivo results. Thus, a more extensive biological characterization can be required for a potential clinical application. 

Currently, there are some types of cancer that were widely studied (e.g., lung, cervical, breast cancer; Table 1 and Table 2). However, other malignancies should be explored, since they have been on a steady rise over the years, such as head and neck cancer, which can be an interesting target to be tested with this type of therapy.

## Figures and Tables

**Table 1:** G4 sequences strategies and biological assays used to analyze the anticancer potential.

G-Quadruplex Sequences	Anticancer Strategy	Cancer Type	Biological Assays	Reference
Aptamers				
AS1411	TMPyP4	Breast cancer	In vitro	[29]
	C_8_	Cervical cancer	In vitro	[35]
	ZnPc1-4	-	-	[36]
AS1411-ICG	C_8_	Melanoma	In vitro and in vivo	[37]
AS1411	NHC-Au(I)	Breast, cervical and prostate cancer	In vitro	[38]
AS1411-dsDNA	Doxorubicin	Breast cancer	In vitro and in vivo	[39]
DTN-AS1411	-	Lung cancer	In vitro and in vivo	[40]
4-arm AS1411/RNA construct	-	Breast cancer	In vitro	[41]
AT11	C_3_, C_5_, C_8_	Cervical cancer	In vitro	[7]
	ZnPc1-4		In vitro	[36]
AT11-B0	C_3_, C_5_, C_8_	Cervical cancer	In vitro	[7]
	ZnPc1-4		In vitro	[36]
AT11-L0	C_3_, C_5_, C_8_	Cervical cancer	In vitro	[6]
	ZnPc1-4		In vitro	[36]
APTA-12	Gemcitabine	Pancreatic cancer	In vitro and in vivo	[43]
	Gemcitabine and doxorubicin	Breast cancer	In vitro	[44]
pre-miR-149	C_8_, C_8_-NH_2_	Prostate Cancer	In vitro	[45]
AIR-3	5-FUdR	hIL-6R-presenting cells	In vitro	[47]
Non-aptamers				
G-quadruplex–Sgc8	TMPyP4	Acute lymphoblastic leukemia	In vitro	[55]
G - quadruplex–TD05	TMPyP4	Burkitt’s lymphoma	In vitro	[55]
AptG15	Methylene blue	Acute lymphoblastic leukemia	In vitro	[56]

**Table 2:** G4-based nano-particles and biological assays used to analyze the anticancer potential.

G-Quadruplex-Based Nano-Particles	Anticancer Strategy	Cancer Type	Biological Assays	Reference
Liposomes				
NCL-aptamer-(HSPC/Cholesterol/mPEG2000–DSPE)	Cisplatin	Breast cancer	In vitro	[65]
	Doxorubicin		In vitro and in vivo	[66]
AS1411/TGN- (PEG-PCL)	Docetaxel	Glioma	In vitro and in vivo	[68]
AS1411- (PEG–PLGA)	Paclitaxel	Glioma	In vitro and in vivo	[69]
AS1411-(PEG/Cholesterol/DOPE)	anti-BRAF siRNA	Melanoma	In vitro and in vivo	[70]
AS1411-(DOPE/SM/Cholesterol/DSPE-PEG2000/DDAB)	Paclitaxel and anti-PLK1 siRNA	Breast cancer	In vitro and in vivo	[71]
AS1411-(PC/Cholesterol/DSPE-PEG)	5-FU	Basal Cell Carcinoma	In vitro	[72]
AS1411-(DPPC/Cholesterol/PEG2000-DSPE) loaded with ABC	Doxorubicin	Breast cancer	In vitro and in vivo	[73]
AS1411-(PC/Cholesterol) coated with MnO2 nano-sheets	HMMEr and ACF	Ovarian cancer	In vitro and in vivo	[75]
AS1411-(α-Gal glycolipids/Phospholipids/Cholesterol)	-	Breast cancer	In vitro	[76]
Micelles				
AS1411-(TPGS-b-PBAE)	Paclitaxel	Ovarian cancer	In vitro and in vivo	[78]
AS1411-(Pluronic F127/β-CD-linked PELA)	Doxorubicin	Breast cancer	In vitro and in vivo	[80]
AS1411-(CPU)	Anti-TLR4 siRNA and doxorubicin	Lung cancer	In vitro and in vivo	[81]
Other lipidic structures				
cMet/lipidated GC-rich DNA hairpin/2′,6′-dimethylazobenzene moieties	Doxorubicin	Lung cancer	In vitro	[85]
AS1411-(Perfluorocarbon/DPPC/DSPE-PEG2000)	Thymoquinone	Breast cancer	In vitro	[86]
Ap–CIS–NPs and Ap-anti-miR-21-NPs	Cisplatin and anti-miR-21	Ovarian cancer	In vitro	[87]
CIS-PCL NPs and LNA-PCL NPs	Cisplatin and anti-miR-214	Ovarian cancer	In vitro	[88]
AS1411-decorated niosomes	Ru(III)-complex HoThyRu	Cervical cancer	In vitro	[89]
Dendrimers				
AS1411- pegylated PAMAM	Camptothecin	Colorectal cancer	In vitro and in vivo	[90]
AS1411- PAMAM modification with 10-bromodecanoic acid (10C) and 10C-PEG	Bcl-xL shRNA	Lung cancer	In vitro	[91]
AS1411-PAMAM grafted with persistent luminescence nano-particles	Doxorubicin	Cervical cancer	In vitro and in vivo	[92]
MUC1/AS1411-Dendrimer(G_3_)	Epirubicin	Breast cancer/Colon carcinoma	In vitro and in vivo	[93]
Gold nano-particles				
AS1411- gold AuNPs (5 nm)	-	Breast cancer	In vitro and in vivo	[98]
AS1411- AuNPs (13 nm)	N-methylmesoporphyrin IX	Breast cancer	In vitro	[99]
AuNPs@βCD@DRHC	TMPyP4	Hepatocyte carcinoma	In vitro	[100]
AS1411-AuNPs with a bifunctional linker containing a dithiolane and a self-immolative disulfide based structure	Doxorubicin or AZD8055	Breast cancer/Uveal melanoma	In vitro	[102]
Graphene nano-particles				
Self-catalytic GO-He-G4-M@DOX nano-medicine	Doxorubicin	Colon cancer/Cervical cancer/Prostate cancer/Breast cancer	In vitro and in vivo	[62]
Silica nano-particles				
DOX-loaded FA-modified MSP-DNA-TMPyP_4_	Doxorubicin and TMPyP_4_	Liver cancer	In vitro	[63]
Magnetic nano-particles				
TMPyP and DNM and Apt-S8@SPION	Daunomycin and TMPyP_4_	Lung cancer and colon carcinoma	In vitro	[106]
Other types of nano-particles				
DTX/ABC@TSLs@Au-DA	Docetaxel	Breast cancer/Sarcoma	In vitro and in vivo	[110]

## Data Availability

Data is contained within the article.
